# Ferroptosis-associated genes and compounds in renal cell carcinoma

**DOI:** 10.3389/fimmu.2024.1473203

**Published:** 2024-09-27

**Authors:** Chengwu He, Qingyi Li, Weijia Wu, Ke Liu, Xingwen Li, Hanxiong Zheng, Yongchang Lai

**Affiliations:** ^1^ Department of Urology, Shenzhen Shockwave Lithotripsy Research Institute, The Eighth Affiliated Hospital of Sun Yat-sen University, Shenzhen, Guangdong, China; ^2^ Tibet Future Biomedicine Company Limited, Golmud, Qinghai, China; ^3^ Department of Pharmaceutical Management, School of Medical Business, Guangdong Pharmaceutical University, Guangzhou, Guangdong, China

**Keywords:** renal cell carcinoma, ferroptosis, Von Hippel Lindau, targeted therapy, immunotherapy

## Abstract

As the main type of renal cell carcinoma (RCC), clear cell RCC (ccRCC) is often associated with the deletion or mutation of the von Hippel Lindau (VHL) gene, enhancement of glucose and lipid metabolism, and heterogeneity of the tumor microenvironment. VHL alterations in RCC cells lead to the activation of hypoxia-inducible factors and their downstream target vascular endothelial growth factor, and to the reprogramming of multiple cell death pathways and metabolic weakness, including ferroptosis, which are associated with targeted therapy or immunotherapy. The changes in biological metabolites (e.g., iron and lipids) support ferroptosis as a potential therapeutic strategy for RCC, while iron metabolism and ferroptosis regulation have been examined as anti-RCC agents in numerous studies, and various ferroptosis-related molecules have been shown to be related to the metastasis and prognosis of ccRCC. For example, glutathione peroxidase 4 and glutaminase inhibitors can inhibit pyrimidine synthesis and increase reactive oxygen species levels in VHL-deficient RCC cells. In addition, the release of damage-associated molecular patterns by tumor cells undergoing ferroptosis also mediates antitumor immunity, and immune therapy can synergize with targeted therapy or radiotherapy through ferroptosis. However, Inducing ferroptosis not only suppresses cancer, but also promotes cancer development due to its potential negative effects on anti-cancer immunity. Therefore, ferroptosis and various tumor microenviroment-related molecules may co-occur during the development and treatment of RCC, and further understanding of the interactions, core targets, and related drugs of ferroptosis may provide new combination drug strategies for RCC treatment. Here we summarize the key genes and compounds on ferroptosis and RCC in order to envision future treatment strategies and to provide sufficient information for overcoming RCC resistance through ferroptosis.

## 1 Background

Renal cell carcinoma (RCC) accounts for 2.2% of the total tumor incidence and 1.8% of morbidity worldwide, and its incidence is increasing year by year (1). The major subtypes of RCC include clear cell renal cell carcinoma (ccRCC), papillary cell RCC (pRCC), and chromophobe cell RCC (chRCC), with approximately 80% of RCC cases classified as ccRCC (2). Metabolic reprogramming, commonly involving the alteration of oxygen, lipid, iron, and energy metabolism, is associated with RCC tumorigenesis and development (3). von Hippel Lindau (VHL), a well-known tumor suppressor gene, regulates the cell cycle, apoptosis, metabolism, signal transduction, and many other processes, and the VHL mutation rate in patients with ccRCC is as high as 92% (4). The VHL protein produced by the gene can inhibit angiogenesis to suppress tumor growth.

The most important function of VHL is to negatively regulate hypoxia-inducible factor (HIF) to affect fundamental cellular biological processes (5). VHL mutations lead to the loss of normal VHL protein function and inhibit the ubiquitination and degradation of HIF, causing its accumulation. HIF, a transcription factor, upregulates downstream hypoxia response genes such as vascular endothelial growth factor (VEGF) and platelet-derived growth factor (PDGF) (6). In addition to the traditional forms of cell death (i.e., necroptosis and apoptosis), other cell death pathways include autophagy, pyroptosis, and the newly discovered cuprotosis, ferroptosis, and disulfidptosis (7–10). Ferroptosis depends on iron and reactive oxygen species (ROS), and its morphology, biochemistry, and genetics differs from those of apoptosis, necroptosis, and autophagy (11). The three major steps in ferroptosis are the oxidation of polyunsaturated fatty acids (PUFAs), the arising of redox-active iron, and the inactivation of glutathione (GSH) peroxidase 4 (GPX4), resulting in the loss of the capacity to scavenge lipid peroxide (12). Ferroptosis has been linked to a variety of multiple systemic disorders, including circulatory, neurological, and urological diseases, as well as cancer (13–17). Drugs that target it may provide new treatment opportunities for cancers that have failed to respond to traditional therapy (18). In recent years, significant advances have been made in the understanding of the role of ferroptosis in tumor biology and treatment, including the elucidation of crosstalk between ferroptosis and tumor-related signaling pathways and the potential applications of ferroptosis in systemic treatment, radiotherapy, and immunotherapy (19). Moreover, it is becoming more and more popular to use pathological or radiological omics to predict the overall survival of RCC after operation (20). In this review, we comprehensively summarize the mechanisms underlying ferroptosis, discuss novel ferroptosis targets, and evaluate the potential of new ferroptosis-related treatments for ccRCC.

## 2 Molecular mechanisms of ferroptosis

### 2.1 Lipid peroxidation

Lipid metabolism is related closely to ferroptosis, and the accumulation of ROS produced by lipid peroxidation in cells is a central step in ferroptosis involving non-enzymatic (i.e., Fenton) reaction pathways (21–23). PUFAs are crucial components of cell membranes that provide the main substrate for lipid peroxidation in ferroptosis (22–24). Their abundance determines the availability of lipid peroxidation sites, which in turn determines the susceptibility to ferroptosis (25). Under the action of acetyl coenzyme A synthetase long chain family member 4 (ACSL4), PUFAs bind to CoA and are esterified and inserted into the cell membrane by lysophosphatidylcholine acyltransferase 3 (LPCAT3). They are then catalyzed by lipoxygenases (LOXs) and undergo peroxidation at the allylic position to produce and accumulate lipid peroxide, which eventually leads to cell death (26).

Exogenous monounsaturated fatty acids (MUFAs) can replace PUFAs on phospholipids located on the cell membrane or other membrane structures, thereby reducing the sensitivity of these membranes to the deactivation of GPX4 and accumulation of ROS (27, 28). ACSL4 plays an important role in determining the sensitivity of cells (including mesenchymal cancer and ccRCC cells) to ferroptosis, and the reduction of ACSL4 and LPCAT3 expression in cells can reduce the accumulation of substrate for lipid peroxidation, thereby inhibiting ferroptosis (29, 30). In VHL-deficient ccRCC, lipophilic statins have a “synthetic lethal” effect, inhibiting mevalonate pathway biosynthesis (31). Statins reduce the expression of GPX4 by inhibiting HMG-CoA reductase, leading to the production of lipid ROS and induction of ferroptosis (32).

### 2.2 Iron metabolism

Iron is involved in many physiological and pathological processes (e.g., ferroptosis and ferritin phagocytosis), while iron homeostasis is strictly regulated to prevent harmful effects on organs caused by iron excess or deficiency (33). Different cell types use different strategies to capture various iron subtypes for participation in their intracellular metabolic activities (34). Iron metabolism in cells influences the cells’ susceptibility to ferroptosis (35). The human body contains two iron ions: Fe^2+^ and Fe^3+^ (36). Fe^2+^ is absorbed through the intestinal epithelium via divalent metal ion transporter 1 (DMT1) and then transported to the circulation through ferroportin-1 (FPN1) (37). It is oxidated to Fe^3+^ by ceruloplasmin and binds to transferrin (Tf) in the serum (38). Upon recognition by the transferrin receptor TFRC1 on the cellular membrane, Tf enters the cell through endocytosis. After dissociation with Tf in the endosome, Fe^3+^ is reduced to Fe^2+^ by the metalloreductase six-transmembrane epithelial antigen of prostate 3 (STEAP3). DMT1 then mediates the transport of Fe^2+^ from the endosome to the cell labile iron pool (LIP) (39). Ferritin, which has two subunits – ferritin light chain 1 (FTL1) and ferritin heavy chain 1 (FTH1) – can limit ion participation in redox reactions by forming a complex with iron and proteins (40).

Nuclear receptor coactivator 4 (NCOA4) binds to ferritin and mediates its delivery to autophagosomes (41). When autophagosomes fuse with lysosomes, ferritin is degraded and iron is released. Free Fe^2+^ participates in a series of physiological cell processes, including the generation of ROS through Fenton reactions, which further promotes lipid peroxidation (42). The down-regulation of NCOA4 in cells inhibits ferritin autophagy and ferroptosis, whereas NCOA4 overexpression increases ferritin degradation and promotes ferroptosis, suggesting that NCOA4 is involved in the interaction between autophagy and ferroptosis (43). In addition to participating in Fenton reactions, iron plays roles in other cellular metabolic pathways, such as by being transported to the mitochondria for the synthesis of heme or iron-sulfur (Fe-S) clusters; thus, iron participates in the regulation of iron-mediated cell death in an environment-dependent manner (44).

### 2.3 Ferroptosis defense mechanisms

#### 2.3.1 System X_c_^–^/GSH/GPX4 axis

The System X_c_^–^/GSH/GPX4 axis, DHODH–ubiquinol (CoQH_2_) system, FSP1–CoQ10 axis, GTP cyclohydrolase-1 (GCH1)–tetrahydrobiopterin (BH4) axis, and sex hormones can inhibit ferroptosis, and the inhibition of related molecular pathways is an important strategy to induce ferroptosis in tumor cells (45). System X_c_^–^ is a heterodimeric amino-acid transporter located on the cell membrane and consisting of two subunits: SLC3A2 and SLC7A11 (46). SLC7A11 is the primary functional subunit, responsible for cystine transport into cells (47). Glutamate and cysteine are synthesized into gamma-glutamylcysteine by gamma-glutamylcysteine synthetase (GCL) and then combined with glycine by GSH synthase to synthesize GSH (48). GPX4 reduces lipid peroxides to lipid alcohols using GSH, thereby reducing the toxic accumulation of peroxides and inhibiting ferroptosis (49). The catalytic cycle of GPX4 involves a “ping-pong” mechanism, with alternation between active enzymatic sites in oxidized and reduced states (50). RCC has a metabolic weakness, and GPX4 inhibitors can promote its sensitivity to metabolic reprogramming (51). Common ferroptosis inducers (FINs) act primarily on the System X_c_^–^/GSH/GPX4 axis and can be roughly divided into four classes based on their targets: class-1 FINs such as erastin inhibit SLC7A11, class-2 FINs such as RSL3 and ML162 inhibit GPX4 enzyme activity, class-3 FINs such as FIN56 deplete GPX4 and CoQ10, and class-4 FINs induce lipid peroxidation (52).

#### 2.3.2 Other ferroptosis-associated axes

Although GSH/GPX4, ACSL4, and PUFA are the main factors required for ferroptosis, exogenous oxygen radicals generated by photodynamic therapy (PDT) can directly peroxidize PUFAs and initiate lipid autoxidation, triggering ferroptosis-like cell death by means independent of LOXs and ACSL4 (53). Sebastian Doll discovered that FSP1/Q10 is a non–GSH-dependent inhibitor of ferroptosis (54); carnitination recruits FSP1 to the plasma membrane, where it acts as a redox enzyme, reducing CoQ10 and producing lipophilic radical-trapping antioxidants (RTAs) that prevent lipid peroxide accumulation.

NFE2L2/NRF2 defends against oxidative stress in cells, and various ferroptosis-related proteins, including those related to cellular iron metabolism and GSH metabolism, are its targets (55). NRF2 can upregulate the System X_c_^–^/GSH/GPX4 axis, including SLC7A11, GSH synthase, and GPX4, which inhibits ferroptosis (56). NRF2 expression also induces the expression of NQO1, heme-oxygenase 1 (HO1), and FTH1, affecting intracellular iron metabolism, and these processes can be inhibited by p62 activation (57). Glutaminase inhibitors inhibit pyrimidine synthesis and increase the ROS level in VHL-deficient RCC cells, and the PARP inhibitor olaparib and glutaminase inhibitors synergically inhibit the growth of these cells *in vivo* and *in vitro* (58).

A group of genes that antagonize iron-dependent cell death, including GCH1 and its metabolic derivatives BH4/dihydrobiopterin (BH2), has been identified, and BH4/BH2 synthesis-induced lipid remodeling have been found to inhibit ferroptosis by selectively preventing the consumption of two polyunsaturated acyl-tailed phospholipids (59). DHODH, discovered in 2021, inhibits mitochondrial ferroptosis by reducing ubiquinone to CoQH_2_ (a radicular-trapping antioxidant with anti-ferroptotic activity) (60). Mitochondria-localized GPD2 oxidizes glycerol-3-phosphate (G3P) and also reduces ubiquinone to CoQH_2_, supporting the hypothesis that G3P in mitochondria also acts as an RTA to inhibit ferroptosis (61).

#### 2.3.3 Regulation of ferroptosis and other types of cell death by p53

p53 is an important tumor suppressor that functions as a transcription factor (62). During cellular stress, p53 activates and regulates cell cycle arrest and cellular senescence or apoptosis pathways, and plays roles in tumor suppression (63). Approximately 50% of patients with cancer have a p53 gene mutation that leads to the inactivation of wild-type p53 (64). Recent studies have found that p53 can promote or inhibit cell ferroptosis through various pathways, and that this activity appears to be highly context dependent, possibly depending on cell or upstream and downstream signaling specificity (65). The target genes of p53 involve the canonical ferroptosis pathway of GSH metabolism (e.g., SLC7A11), iron metabolism (TfR1 and SLC25A28), lipid metabolism, ROS generation, several oxidation/reduction related proteins, and the non-canonical ferroptosis pathway (66, 67). Calcium-independent phospholipase A2β (iPLA2β)-mediated lipid peroxidation detoxification is sufficient to inhibit p53-driven ferroptosis under ROS-induced stress and acts as a major ferroptosis suppressor independent of GPX4, suggesting that iPLA2β is a promising therapeutic tumor target (68). Thus, p53 promotes ferroptosis in most cases, but inhibits it in a few cases (66).

In addition to apoptosis, p53 regulates other non-canonical forms of cell death, including ferroptosis, necroptosis, autophagic cell death, and pyroptosis (69). The traditional view suggests that different cell death signaling pathways are independent of each other, but recent studies have shown that these pathways are closely connected and can cross-regulate each other (10). p53 is activated by the apoptosis-stimulating protein to trigger cell apoptosis, a process that is inhibited by the inhibitor of the apoptosis-stimulating protein of p53 (iASPP), which promotes tumor growth (70). iASPP inhibits ferroptosis through the Nrf2/HIF-1/TF signaling pathway, and its overexpression induces chemoresistance in human tumor cells (71).

The p53-upregulated modulator of apoptosis (PUMA) is a downstream target of p53 that mediates cell apoptosis (72). Ferroptotic agents induce endoplasmic reticulum stress to promote PUMA expression through the PERK-eIF2α-ATF4-CHOP pathway (73), but this form of PUMA does not lead to cell apoptosis. PUMA mediates cell apoptosis in response to the tumor necrosis factor (TNF)-related apoptosis-inducing ligand and ferroptosis inducers, and p53 may regulate ferroptosis and apoptosis as a key factor in the crosstalk of the two death mechanisms through PUMA regulation (74). Different conditions or external stimuli may lead cells to different death pathways, with single-type or mixed-type pathways dominating, although mixed-type cell death appears to be more common than single-type death due to the sharing of common regulatory factors and signaling molecules (75). p53 plays key roles in inducing different forms of cell death, but the mechanism by which it prioritizes cell death mechanisms remains unclear. Thus, more research is needed to understand the overall role of p53 in different programmed cell death processes. Ferroptosis-associated targets and molecules are shown in **Table 1**.

**Table 1:** Ferroptosis related targets and molecules.

Common markers:	Characteristic	Key molecules	Ferroptosis inhibitors or ferroptosis promoters
Iron, glutathione, MDA, GPX4, ROS, PTGS2, SLC40A1, LPO, LDH cytotoxicity	Disorders of iron metabolism	Transferrin, Ferroportin, TFR1, ZIP8/14, ROS, DMT1, STEAP3, HSPB1,	Inhibitro: desferrione, desferriamine, delarolex
			Promoter: ferroheme, artemisinin
	Amino acid antioxidant system imbalance	GSH, GPX4, system XC-, P53/SLC7A11	Promotor: sorafenib, RSL3, ML162, salazosulfapyridine, glutamate, cisplatin
			Inhibitro: N-acetyl-l-cysteine
	Lipid peroxidation	ACSL4, LPCAT3, P53-SAT1-ALOX15 pathway	Inhibitros: ferrostatin-1, troglitazone, rosiglitazone, zileuton
			Promotor: FINO2
	VDACs, sulfur transfer pathway, mevalonate pathway, FSP1-COQ10-NADPH pathway, GCH1/BH4/DHFR, ATG5/7-NCOA4 pathway, P62- Keap1-NRF2 pathway	NRF2, NCOA4, P53, FSP1, HMG-coA, DHFR,	Inhibitor: liproxstain-1
			Promotor: erastin, simvastatin, FSEN1, FIN56, iFSP1

FTH1, ferritin heavy chain 1; GPX4, glutathione peroxidase 4; HSPB1, heat shock protein family B (small) member 1; TF, transferrin; TFRC, transferrin receptor; PTGS2, prostaglandin-endoperoxide synthase 2; SLC40A1, solute carrier family 40 member 1; NCOA4, nuclear receptor coactivator 4; LPCAT3, lysophosphatidylcholine acyltransferase 3; ACSL4, acyl-CoA synthetase long chain family member 4; KEAP1, kelch like ECH associated protein 1; ATG5, autophagy related 5; STEAP, six-transmembrane epithelial antigen of the prostate; LPO, lipid peroxide; ALOX, arachidonate lipoxygenase; MDA, malonaldehyde; VDAC, voltage-dependent anion channel; DMT1, divalent metal transporter 1; LIP, labile iron pool; DFO, deferoxamine.

## 3 Ferroptosis in RCC

### 3.1 Ferroptosis signaling pathway and RCC

#### 3.1.1 HIF-iron metabolism pathway involved in ferroptosis and RCC

ccRCC is associated closely with mutations in the VHL gene that lead to the activation of HIF-1α and HIF-2α via a pseudohypoxia mechanism, resulting in the transcription of genes involved in tumor progression (76). The regulation of the VHL–HIF pathway is also related to iron metabolism and ferroptosis in ccRCC, as iron levels are significantly higher in RCC cells and the tumor microenvironment than in normal renal tissue (77), and as HIF-1α mediates the cellular upregulation of TfRC and heme oxygenase (HO) expression, promoting cellular iron accumulation (78, 79). Increased intracellular active iron levels may make VHL-defective RCC cells more susceptible to ferroptosis (80). However, iron chelators have also been shown to inhibit the proliferation of ccRCC cells with VHL dysfunction (81). The inhibition of ISCA2 (a component of the late mitochondrial iron-sulfur cluster assembly complex) reduces the HIF-2α protein level and triggers an iron starvation response, leading to iron/metal overload and contributing to ferroptosis, which is more significant in VHL-defective cells (82). Thus, the genetic and metabolic characteristics of ccRCC cells affect their sensitivity to iron-mediated cell death, but more exploration of the relationship between intracellular iron levels and renal tumor progression is needed.

#### 3.1.2 Ferroptosis-related lipid metabolism and RCC

The acyl-CoA synthetase family member ACLS3 not only regulates the accumulation of lipid droplets in ccRCC and is essential for tumor growth, but also regulates sensitivity to ferroptosis in a manner dependent on the composition of exogenous fatty acids: both functions are implicated in the treatment of ccRCC (83). The inhibition of fatty acid metabolism via HIF increases intracellular lipid storage, and subsequently the sensitivity to iron-mediated cell death (84). The inhibition of chemerin increases intracellular PUFA levels and decreases MUFA levels, leading to impaired mitochondrial respiratory chain function and CoQ10 and CIV down-regulation, resulting in more ROS generation and the induction of iron-mediated cell death (85). In addition, acyl-CoA thioesterase 8 also can affect ferroptosis, but may exert discordant actions in ccRCC tumorigenesis (86).

#### 3.1.3 GSH/GPX4 pathway and RCC

The System X_c_^–^/GSH/GPX4 axis is involved in ferroptosis inhibition (45). ccRCC cells are highly sensitive to the consumption of glutamine or cysteine, and the use of erastin, L-buthionine-S, or R-sulfoximine (BSO) can induce cell death by inhibiting GSH synthesis in ccRCC cells in a concentration-dependent manner (87).

Inactivating mutations of KDM5C, commonly found in patients with ccRCC, alter glycogen metabolism, leading to increased NADPH and GSH levels and thereby inhibiting ferroptosis; thus, glycogen metabolism in KDM5C-deficient ccRCC is a therapeutic target for the induction of ferroptosis (88).

Metallothionein 1G (MT1G), which is related closely to GSH reductase, is upregulated in ccRCC cells and may inhibit ferroptosis by regulating GSH consumption (89). Kruppel-like factor 2 (KLF2) mediates the transcriptional suppression of GPX4 expression, thereby regulating ferroptosis and inhibiting ccRCC cell migration and invasion (90). The overexpression of CX3CL1, a chemokine associated with the immune infiltration of tumor cells, leads to the downregulation of GPX4, thereby inhibiting the GSH/GPX4 axis (91).

#### 3.1.4 The hippo and other ferroptosis-related pathway and RCC

The knockdown of MIT domain-containing protein 1 down-regulates TAZ (a downstream signaling factor of the Hippo pathway) and causes the inhibition of SLC7A11 expression, thereby inhibiting cellular cysteine uptake and inducing ferroptosis (92). On the other hand, TAZ promotes cell density mediated ferroptosis of RCC cells via regulating EMP1-NOX4 (93).

**Figure 1:**
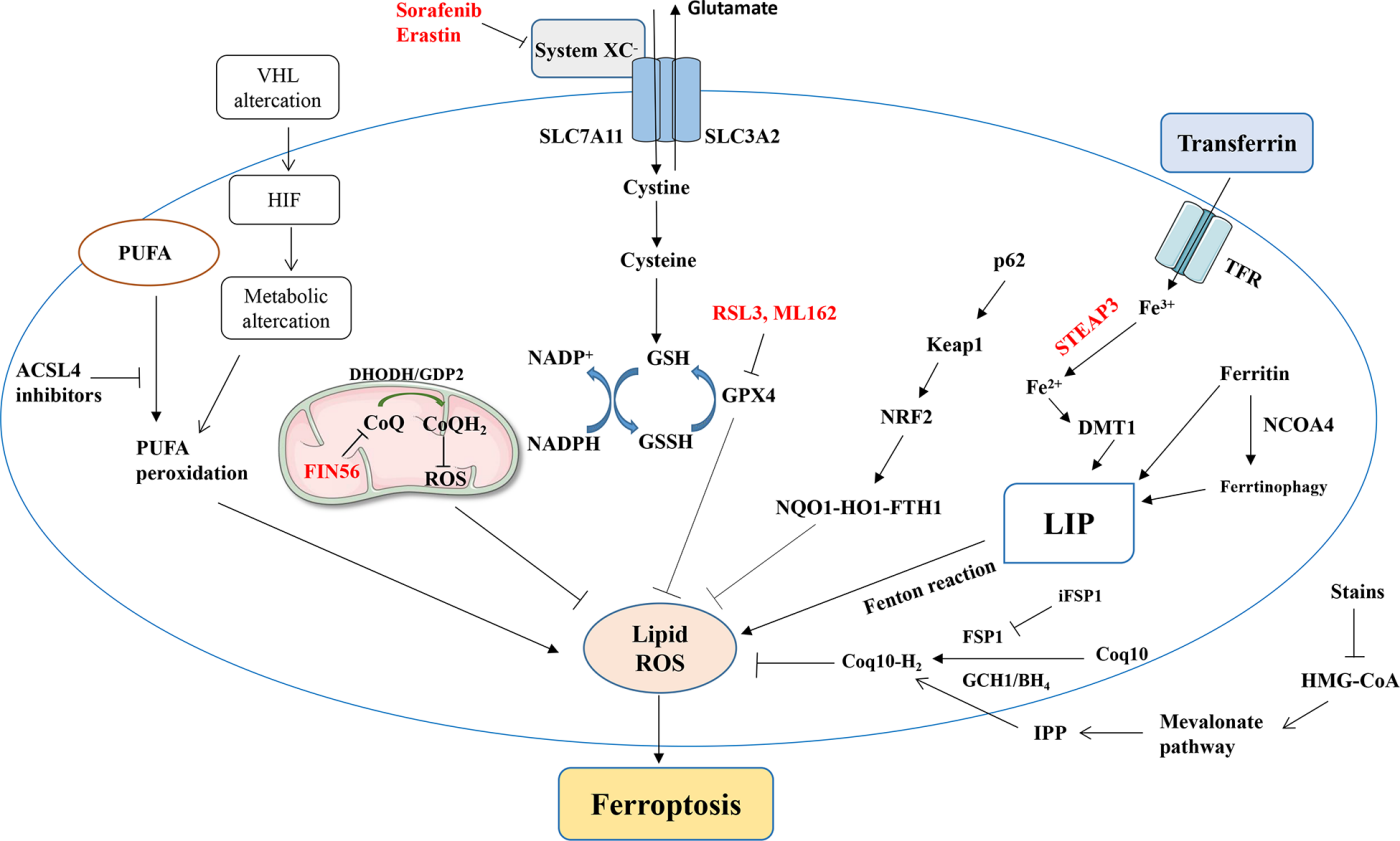
A simplified summary of ferroptosis-related signaling pathways and ferroptosis induced by various factors. NRF2, nuclear factor erythroid 2-related factor; GPx, glutathione peroxidase; GSH, glutathione; PUFA, polyunsaturated fatty acid; ROS, reactive oxygen species; TF, transferrin; TFRC, transferrin receptor; HMGR, HMG-CoA reductase; ACSL4, acyl-coenzyme A synthase long-chain family member 4.

Succinate dehydrogenase (SDH) is significantly down-regulated in ccRCC tissue, leading to the activation of cell antioxidant genes and thereby conferring resistance to ferroptosis (94). Suppressor of variegation 3-9 homolog 1 (SUV39H1)-encoding histone H3 lysine-9 methyltransferase is often upregulated in ccRCC tumors, and its expression level is an independent risk factor affecting prognosis; its deficiency regulates the H3K9me3 state of the dipeptidyl peptidase-4 (DPP4) gene promoter, causing DDP4 upregulation and ferroptosis (95). Ferroptosis-associated signaling pathways are summarized in **Figure 1**.

### 3.2 Ferroptosis-associated genes in ccRCC

Ferroptosis-related signature and diverse programmed cell death was used to predict the prognosis and drug sensitivity of various type of cancer (96, 97). Ferroptosis-related genes (FRGs) that are abnormally expressed in ccRCC cells relative to normal cells include CARS1, CHAC1, CD44, STEAP3, AKR1C1, DPP4, SLC7A11, SLC1A5, and NCOA4 (98–101). A survival model for patients with ccRCC based on four of these genes (CD44, DPP4, NCOA4, and SLC7A11) demonstrated excellent prognostic efficacy, and risk scoring was an independent factor for prognosis prediction (102).

According to FRG expression profiles, patients can be divided into groups with different degrees of immune infiltration and PD-L1 expression. The CARS level correlates positively with PD-L1 expression and may induce the trans-sulfur pathway, leading to cysteine deprivation and ferroptosis inhibition (103). Based on the mRNA expression profiles of 57 FRGs, a ferroptosis scoring system reflecting different prognoses and degrees of immune cell infiltration for ccRCC can be created to predict the sensitivity of patients to different treatments, illustrating that ferroptosis may involve multiple complex tumor microenvironments (98). However, many FRGs identified by bioinformatics remain to be verified experimentally. The FRGs identified by bioinformatics or by experiments in ccRCC are shown in **Table 2**.

**Table 2:** The FRGs identified by bioinformatics or by experiments in ccRCC.

Gene Symbol of ferroptosis-related prognosis model in ccRCC	The FRGs validated by experiment in ccRCC	Document No. (PMID No.)
DPEP1, NOX4, MT1G, GLS2, GLRX5, TIMP1, CA9, CDCA3, CYBB	The GLS2 expression and function	36230613
CD44, BID, TRIB3, TAZ	The CD44 expression and function	35155251
Up-regulated FRGs (CDKN1A, HSPA5, EMC2, SLC7A11, MT1G, HSPB1, FANCD2, SLC1A5, RPL8, LPCAT3, DPP4, CARS); Down-regulated FRGs (NFE2L2, GPX4, CISD1, FDFT1, NCOA4, GLS2, CS, ATP5MC3, ACSL4).	The CARS expression	34291048
CD44, DPP4, NCOA4, SLC7A11	The CD44, DPP4 and SLC7A11 expression.	34370716
High-risk genes (CARS1, MT1G, ACACA, FADS2); Low-risk genes (AKR1C1, HMGCR, CRYAB, NCOA4).	The CARS1, CRYAB and FADS2 expression.	34249761
NCOA4	The NCOA4 expression	33402128
HAMP	Without experiment	36673667
CARS1, CD44, FANCD2, HMGCR, NCOA4, SLC7A11, ACACA	Without experiment	34178024
ACADSB	Without experiment	35096573
CARS, NCOA4, FANCD2, HMGCR, SLC7A11	Without experiment	32688345
CARS, CD44, DPP4, GCLC, HMGCR, SAT1, HSPB1, NCOA4, PHKG2, GOT1, HMOX1	Without experiment	33996565

By binding with KEAP1, DPP9 promotes the stability of NRF2 and overcomes oxidative stress, and the accumulation of DPP9 can lead to the hyperactivation of the NRF2 pathway and inhibit ferroptosis, thereby driving ccRCC development and sorafenib resistance (68). Targeting the Nrf2/Heme oxygenase-1 pathway can significantly increase oxidative stress in RCC and promote apoptosis and death of cancer cells (104), while Heme oxygenase-1 has a dual role in ferroptosis induction (105). The farnesyl-diphosphate farnesyltransferase gene has the potential to be used as a biomarker for the ccRCC diagnosis and to treat ccRCC by regulating ferroptosis (105).

### 3.3 Ferroptosis-associated lncRNAs in ccRCC

Long non-coding RNAs (lncRNAs) are longer than 200 nucleotides, lack protein translation ability, and are expressed specifically in some tumor tissues (106). They regulate gene expression through transcription, translation, protein modification, and RNA–protein complexing (107). In tumor cells, lncRNAs can act as oncogenes or tumor suppressor genes by regulating various signaling pathways (108). LncRNAs act as biomarkers of or therapeutic targets for cancer based on their specific signaling of certain cell states (109–112). Recent studies have shown that the lncRNA 00312/miR-34a-5p/ASS1 axis reduces proliferation and invasion and promotes apoptosis in RCC (113), and that the SNHG12/SP1/CDCA3 ferroptosis-related long non-coding RNA (FRIncRNA) axis promotes RCC progression and sunitinib resistance, thereby serving as a new target for the treatment of sunitinib-resistant RCC (114).

The expression patterns of FRIncRNAs can be used to predict prognoses, and a risk model constructed using patient-specific FRlncRNAs has shown good diagnostic and prognostic value (115). The p53 signaling pathway, TNF-mediated signaling pathways, cytokine-cytokine receptor interaction, and helper T-cell immune response signaling pathways are associated with high-risk FRlncRNAs (116). The expression characteristics of lncRNAs are related to the immune infiltration of ccRCC, and immune checkpoint gene expression differs significantly between low-risk and high-risk groups, which may be related to patient sensitivity to immune and targeted therapies (117).

### 3.4 Ferroptosis-associated genes in pRCC and chRCC

As a key regulator of ferroptosis, SLC7A11 is up-regulated in ccRCC, chRCC, and pRCC, and its overexpression is associated with their poor prognosis and immune cell infiltration (118). SLC7A11, HMOX1, and MT1G were the 3 FRGs associated with the prognosis of adrenocortical carcinoma (ACC), KICH, KIRC and KIRP, while the SLC7A11 is a prognostic risk factor for above 4 different renal tumors, and high expression of MT1G increases the prognostic risk of ACC, ccRCC and chRCC (119).

GCLC, HSBP1 and ACSF2 are independent prognostic FRGs in pRCC (120, 121). The FRGs features composed of CDKN1A, MIOX, PSAT1 and RRM2 are independent prognostic indicators of pRCC, and have a good predictive ability for the prognosis of pRCC patients (122).

For chRCC, targetable pathways for innate lymphoid cell/IL-15 and cysteine homeostasis/ferroptosis have recently been discovered (123). TFRC and SLC7A11 are highly expressed FRGs in chRCC tissues and can predict the clinicopathological features and prognosis of chRCC (124). Gamma-glutamyltransferase 1 (GGT1), a key enzyme in glutathione homeostasis, is significantly down-regulated in chRCC compared with normal kidneys, while overexpression of GGT1 can inhibit the chRCC cells proliferation, and inhibit the cystine uptake and GSH levels (125).

## 4 Treatment of RCC

### 4.1 Targeted therapy and ferroptosis

ccRCC is the most common type of kidney tumor, and traditional treatment methods for it include surgical resection, chemotherapy, and radiation therapy (126). As ccRCC is often associated with VHL gene mutations, targeted drug therapies have show greater efficiency and safety than traditional therapies which offer new hope for kidney cancer patients (127). However, VHL deletion alone is usually not sufficient to drive tumor development; the co-deletion of VHL and one (or more) of the common and important drivers of ccRCC (polybrominated 1 gene, BRCA1-associated protein 1, and SET domain 2) is often required (128, 129). In addition to the VHL-HIF-VEGF/vascular endothelial growth factor receptor (VEGFR) pathway, VHL mutations affect signaling regulation in the mTOR and epidermal growth factor receptor (EGFR) pathways, promoting tumor growth (130). HIF signaling changes the extracellular matrix, regulates the tumor immune response, increases angiogenesis, and affects the tumor microenvironment, altering the metabolism of tumor and stromal cells and increasing tumor invasiveness and metastasis (131). Traditional radiation treatment and chemotherapy are almost completely ineffective for RCC, and the development of targeted drugs against VEGFR and its ligand VEGF is considered to be the primary therapeutic intervention required for patients with advanced RCC (132). However, there is increasing evidence that induction of ferroptosis can reverse tumor chemotherapy resistance (133). Bevacizumab was the first anti-angiogenic drug used in patients with RCC, followed by VEGFR inhibitors such as sunitinib, sorafenib, pazopanib, cabozantinib, and axitinib (101). Beyond that, targeted therapies for DNA damage repair, tumor microenviroment remodeling and tumor cell metabolism reprogramming show potential (134, 135). In March 2021, the FDA approved tivozanib, a potent selective VEGFR-TKI, for the third - and fourth-line treatment of relapsed refractory RCC (136). ccRCC often involves the reprogramming of glucose and lipid metabolism and the tricarboxylic acid cycle, as well as the alteration of tryptophan, arginine, and glutamine metabolism, all of which may provide potential biomarkers or treatment strategies (137). However, resistance to anti-angiogenic therapy often develops in the clinical treatment of ccRCC due to the crosstalk between VEGFR and other tyrosine kinases or downstream pathways; the use multi-target (e.g., HIF) inhibitors and combination strategies carries more potential for the treatment of ccRCC (138).

Crosstalk between different cell death pathways may also be related to resistance to targeted therapy, and the induction of different types of cell death, such as ferroptosis, through targeted therapy has become a new strategy in kidney cancer treatment (139). The metabolic reprogramming properties of ccRCC give it cysteine- and GPX4-dependent sensitivity to ferroptosis, and cysteine deprivation and GPX4 inhibition can induce cell death (51, 80). In addition, some commonly used targeted drugs have been found to induce tumor ferroptosis. For example, sorafenib, used commonly in the treatment of liver and kidney cancers, has been reported to have a similar ferroptosis induction effect for RCC cells to that of erastin (140). Disulfiram/copper can synergistically inhibit the RCC cells growth and induce ferroptosis with sorafenib (141). The accumulation of DPP9 leads to the overactivation of NRF2 and its transcriptional target SLC7A11, which inhibits ferroptosis and induces the resistance of ccRCC cells to sorafenib (142). In addition to sorafenib, sunitinib and glycogen synthase 1 inhibitors can produce synthetic lethal effects in RCC cells (143). Inhibition of fibroblast growth factor receptor 4 significantly reduces glutathione synthesis, leading to excessive ROS production and unstable iron pool accumulation, which triggers ferroptosis to overcome drug resistance of HER2-positive breast cancer (144). Everolimus, an mTOR signaling pathway inhibitor typically used in tumor-targeted therapy, inhibits the mTOR–3EBP4 axis, reduces GPX4 expression, and acts synergistically with RSL3 or erastin to induce ccRCC cell ferroptosis (145).

Activation of ferroptosis by IL-6 is may provide a new strategy for the treatment of advanced RCC and new ideas for TKIs resistance (146). Lysine acetyltransferase 7 inhibits ccRCC by regulating cell cycle and ferroptosis sensitivity (147). As a key enzyme in the tricarboxylic acid cycle, MDH2 play a non-metabolic role in ccRCC progression by reducing FSP1 protein levels and increasing the ccRCC’s sensitivity to ferroptosis (148). SETD2 catalyzes the interaction of histone H3 lysine 36 trimethylation (H3K36me3) with the ferrochelatase promoter, and knockdown of SETD2 significantly increases erastin sensitivity and promotes ferroptosis in ccRCC (149). With the progress of clinical trials such as KEYNOTE-426, the combined use of immune checkpoint and protein tyrosine kinase inhibitors is now one of the standard treatment options for most patients with metastatic RCC (150).

### 4.2 Immune therapy and RCC

Immune therapies, particularly the use of immune checkpoint inhibitors (e.g., anti-PD-1, anti-PD-L1, and CTLA-4 antibodies; CAR-T cell adoptive immunotherapy; and cancer vaccines) are used commonly and represent significant advances in the treatment of malignant tumors (151).

In addition to the traditional perforin–granzyme and Fas–Fas ligand cell death induction pathways, interferon-γ secreted by CD8+ T cells activated in anti–PD-L1 therapy can down-regulate System X_c_^–^ expression to reduce cystine uptake and induce ferroptosis (152). The release of damage-associated molecular patterns by tumor cells undergoing ferroptosis also mediates antitumor immunity, and immune therapy can synergize with targeted therapy or radiotherapy through ferroptosis (153, 154). USP8 inhibitor combined with ferroptosis inducer can significantly inhibit tumor growth and promote the infiltration of CD8+T cells in the tumor, and further improve the therapeutic effect of PD-1/PD-L1 blocking antibodies (155). Activation of ferroptosis and inhibition of myeloid suppressor cells (MDSC) combined with anti-PD-1 monoclonal antibody can greatly improve the survival rate of liver tumor mouse models and reduce liver metastasis of other tumors *in situ* (156). Intermittent deprivation of methionine in the diet significantly enhances tumor sensitivity to ferroptosis and collaborates with PD-1 antibodies to inhibit tumor progression and enhance T-cell-mediated anti-tumor immune response (157). However, ferroptosis may be a double-edged sword for tumor cells, which can enhance not only the function of anti-tumor immune cells but also the tumor-promoting response of immunosuppressive cells under certain circumstances (158). Thus, ferroptosis interacts with the immune system, and the combined use of immune and ferroptosis-targeting therapy for RCC is anticipated in the future.

**Figure 2:**
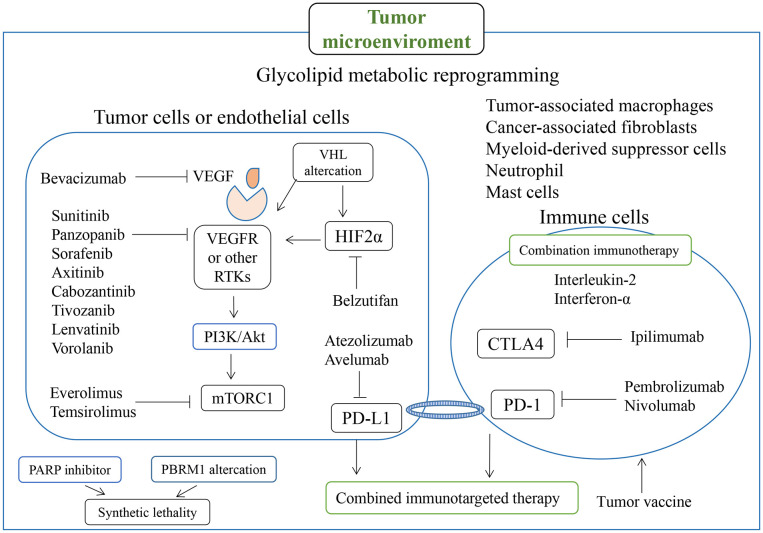
Molecules involved in RCC-related drugs and targets, particularly for targeted therapy and immunotherapy, and regulation associated with the tumor microenvironment.

PD-1 is highly expressed in infiltrating lymphocytes in various tumors, and PD-1 ligands are often upregulated on the surfaces of tumor cells and inhibit the local antitumor immune response mediated by T cells (159). PD-L1 was found to be expressed in 24.2% of RCC tumors, and the level of PD-L1 expression correlated positively with the risk of patient death (160). Recently, the combined use of targeted drugs and immune therapy has yielded favorable outcomes, and combined anti-angiogenic therapy and anti-PD-1/PD-L1 pathway targeting has been shown to improve patients’ clinical outcomes (161). By promoting dendritic cell differentiation, reducing Treg cell aggregation, increasing cytotoxic T-cell abundance, and increasing tumor susceptibility to immune-mediated cytotoxicity, targeted therapy and tumor immunity are believed to interact with each other (162–165). In addition to the use of TKIs combined with PD-1/PD-L1 inhibitors, dual immunotherapies (ipilimumab with nebuliumab and atrilizumab with bevacizumab) are currently used for the first-line treatment of advanced renal cancer (166). RCC-associated drugs, targets, and tumor microenvironment associations are summarized in **Figure 2**.

### 4.3 Nanotherapeutic and natural compound therapy for RCC

Nanoparticles (NPs) are a new form of cancer treatment delivery that overcome some limitations of traditional anti-cancer drugs and can induce various cancer cells to undergo ferroptosis through various means (167, 168). Due to the unique biophysical and chemical properties of metal-based nanomaterials after entering tumor cells, they can also induce ferroptosis and enhance the ability of catalytic therapy (169). Iron oxide (Fe_3_O_4_) NPs produce toxic heat effects and induce ferroptosis via ACSBG1, an acyl-CoA synthetase that acts as a key pro-ferroptotic factor in this tumor-specific process (170). MIL-101 (Fe)@RSL3 NPs deliver iron ions and RSL3 to ccRCC cells, which generate large amounts of ROS through Fenton reactions, leading to the accumulation of LOX, and as GPX4 activity is inhibited by RSL3, this combination induces cell death by ferroptosis (171). Although nanotherapy has many advantages, some problems remain: the complexity and heterogeneity of tumor biology lead to uncertainty about its efficacy, as the interaction of NPs with this biology is not yet clear, and challenges related to NP cost, chemical processes, manufacture, and control persist (172, 173). Furthermore, current research is mostly limited to animal models, and gap between research and clinical application remains (172, 173).

**Figure 3:**
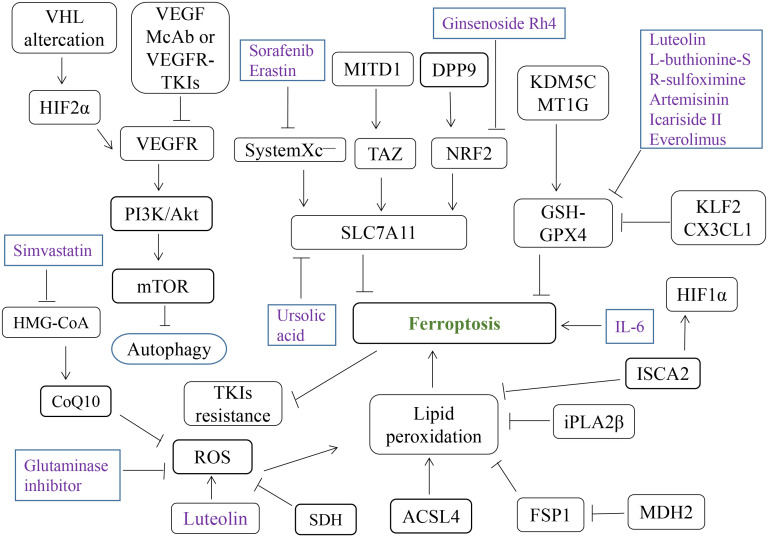
Various ferroptosis-related compounds and genes involved in RCC. ROS, reactive oxygen species; TKIs, tyrosine Kinase Inhibitors; McAb, monoclonal antibody.

Natural compounds are used widely in the treatment of various cancers, including RCC (174). Luteolin, a natural flavonoid found widely in fruits and vegetables, promotes ferroptosis via GSH depletion and ROS accumulation through Fenton reactions (175). Icariside II upregulates miR-324-3p and inhibits GPX4 expression to induce ferroptosis in ccRCC (176). Artemisinin inhibits resistant ccRCC growth by downregulating GSH and GPX4 expression (177). Curcumin increases ADAMTS18 gene expression and restores ccRCC sensitivity to sunitinib, and this regulation can be reversed by ferroptosis inhibitors, indicating that curcumin mediates ferroptosis in ccRCC through ADAMTS18 (178). Ursolic acid and sorafenib can synergically inhibit various tumor, which may be related to the induction of Mcl-1-related apoptosis and SLC7A11-dependent ferroptosis (179). Erianin can induce ferroptosis of RCC stem cells by promoting N6-methyladenosine modification of ALOX12/P53 mRNA, thus may play a role in RCC treatment (180). Sanguinarine reduces the expression of GPX4 and increases the expression of ACSL4, thereby inducing the ferroptosis of RCC cells (181). Ginsenoside Rh4 can increase the sensitivity of RCC cells to ferroptosis by inhibiting the expression of NRF2 signaling pathway and antioxidant enzymes (182). Overall, the natural compounds associated with ferroptosis provide numerous new structural bases for the treatment of RCC. Various compounds and genes targeting ferroptosis in RCC are summarized in **Figure 3**.

## 5 Conclusion and prospects

Many studies have shown that the expression of ferroptosis-related molecules is related to the metastasis and prognosis of ccRCC, the main type of RCC. ccRCC is often associated with the deletion or mutation of the VHL gene, enhancement of glucose and lipid metabolism, and heterogeneity of the tumor microenvironment. VHL changes in RCC lead to the activation of HIF and its downstream target VEGF, and to the reprogramming of multiple cell death pathways and metabolic weakness, including ferroptosis, which are associated with targeted therapy. The changes in biological metabolites (e.g., iron and lipids) support the potential use of ferroptosis, a unique form of cell death, as a therapeutic strategy for RCC. GPX4 and glutaminase inhibitors can inhibit pyrimidine synthesis and increase ROS levels in VHL-deficient renal carcinoma cells, and the PARP inhibitor olaparib and glutaminase inhibitors can synergistically inhibit the growth of these cells *in vivo* and *in vitro*.

In addition to the above-mentioned regulatory mechanisms of iron metabolism and ferroptosis, the development of epigenetic modification and ferroptosis associated drugs may be potential new strategies for anti-RCC (183). In addition, crosstalk and interaction occur among different forms of cell death; ferroptosis promotes cell sensitivity to apoptosis, apoptosis can be transformed into ferroptosis under certain conditions, autophagy promotes ferroptosis (184), and ferroptosis regulators interact with cuprotosis. SLC7A11, a key FRG in pan-RCC and a major functional subunit of the system X_c_^–^, which participates in cysteine transport and inhibits cellular ferroptosis by participating in GSH synthesis, seems to have opposite effects on the regulation of disulfidptosis and ferroptosis, and further exploration of both of these effects is needed (185). In summary, ferroptosis is a distinct form of cell death, but different types of cell death may co-occur during the development and treatment of RCC. In addition, interaction and crosstalk may occur within the signaling pathways involved in ferroptosis. Further understanding of these interactions may provide new combination drug strategies for targeted therapy and immunotherapy of RCC.
